# Intelligence

**Published:** 1828-01

**Authors:** 


### INTELLIGENCE.

### MONTHLY REPORT OF PREVALENT DISEASES.

DURING the last month, we have had the usual routine of Rheumatism, Dyspepsia, and Winter Cough, but without any thing deserving of particular notice.

---

*The Physicians' intended Petition to the Legislature.*—IT has been determined that a petition shall be presented to Parliament, early in the ensuing session, on the part of the Doctors of Medicine of the United Kingdom, Graduates of the Scotch and other Universities, with a view of engaging the legislature to emancipate the profession from the shackles which have for centuries been so illegally and unjustly imposed on it by the arrogated professional monopoly of the College of Physicians in London; of which petition the following is an outline :—

1. It is essential to the national welfare that his Majesty's subjects be supplied with medical attendance, of excellence commensurate with the improved state of science, and avouched by competent testimonials.

2. To effect these objects, it is necessary that the profession of physic be so organised as to insure adequate qualifications on the part of practitioners, and afterwards to secure, to individuals so qualified, full power to exercise their talents, in the way most conducive to the public good and to their own interests.

3. On this account, it is incumbent on the legislature to establish, by law, such an arrangement of the profession, in its several branches, as shall insure that all who profess the practice of the art possess the necessary qualifications.

4. The supply of medical aid being left free to accommodate itself to the demand, the welfare of the profession requires that its different branches be protected, both from unqualified intrusion and from unnecessary restrictions.

5. To enable your honorable House to judge of the alterations required for this profession, it appears necessary to inquire into the nature of the divisions of its different branches,—the relative proportion of their numbers to each other, and to the population, in this, as compared with other nations, —as well as the causes and effects of these divisions and proportions respectively.

6. That a feeling very generally prevails that serious grievances do exist in the medical profession, is placed in evidence by the following facts, either officially before your honorable House or otherwise notorious :—Last year it was resolved by the surgeon-apothecaries, or general practitioners, that a petition be presented to Parliament, praying for an inquiry into the existing state of medicine and surgery, so far as regards the general practitioner, &c. It was about the same period resolved, by the members of the surgical profession, that a petition be presented to the House of Commons, for a committee to inquire into the abuses of the College of Surgeons, &c.; and your petitioners now humbly state their grounds for respectfully soliciting a revision of all the laws which regard the several branches of the profession, particularly the physicians' department, and also the laws by which the College of Physicians of London are, or profess to be, governed.

7. Your petitioners presume the more earnestly to solicit the attention of your honorable House to those circumstances, from having observed the hazard of legislating for any single department of a collective profession, without special reference to the interests of all its branches, as well as of the community at large. As an instance in point, they might mention the unsuitable powers unwarily granted to the Society of Apothecaries, by the Act of 1815.

8. Your petitioners now come to their more immediate object, of submitting to your honorable House a general view of the grievances under which the profession and the public labour, as connected with the organization of their own department, which abounds in evils urgently calling for legislative remedy.

9. Upwards of three centuries ago, the physicians of London were constituted a corporation, or college (unum Corpus et Communitas perpetua, sive Collegium perpetuum), principally for the purposes of "restraining and suppressing illiterate, unexperienced, and unlicensed practisers;" to which association were granted all the usual rights and privileges which royal charters confer.

10. It would be well if the spirit, or even the letter, of this charter had been regarded by those who administered its powers; but, unhappily, the course of proceeding adopted by this body, from its earliest formation, has set at naught the laws under which they were to be governed; and, by arbitrary and illegal by-laws, they have superseded the authority of the statutes, introducing practices to which these statutes afford no sanction,—excluding a large portion of the profession from rights to which the charter and statutes give them claim,—creating distinctions utterly at variance with all that the law enjoins, —and so limiting the College, as, without any legal authority whatever, to exclude from its pale all who do not derive their qualifications from the medical degree of Oxford or Cambridge.

11. Were this power even conferred by statute, it would, at the present day, be an oppression as absurd as it is unjust; for, in the course of events, other medical schools in Britain have risen to an eminence far surpassing what Oxford or Cambridge could ever boast of; and the law, if it existed, would present the solecism of conferring the highest medical honours upon those who least deserved them: nay, of wholly limiting them to this favored few; all other physicians, however eminent or capable, being absolutely excluded.

12. These regulations operate with peculiar severity and injustice on physicians of the catholic and dissenting persuasions, who are, under all circumstances, excluded from the fellowship.

13. The law, however, gives no such power; for your petitioners seek in vain, either in the charter or statutes, for any grounds on which the proceedings pursued by this College can be justified.

14. So entirely has the reputed College of Physicians departed from the legal authorities from which it springs, that the whole discipline of the College, and even its very denomination, differ essentially from what the law has directed; so that, were the present College required to prove its identity with that of Henry VIII., your petitioners are persuaded they would be unable to establish the fact.

15. In the whole course of proceeding followed by this College, your petitioners can discern no motive having for its object the public good, or the advantage of the profession; its unvarying tendency being to establish within the profession an odious monopoly,—giving rank exclusively to those who can show no claim to professional superiority; attempting to make the largest portion of British physicians outcasts and aliens in their own country; and, by the influence of these signal abuses, degrading the whole profession, and exposing it to the encroachments which the subordinate departments have so successfully made on it.

16. Of these encroachments, however glaring, your petitioners mean not to complain, nor do they seek protection from them in any exclusive rights or privileges.

17. Your petitioners, however, do seek relief from the unjust and illegal restrictions imposed on them by the College of Physicians, so as to enable them to enter into professional competition with an equal chance of maintaining their own title to public approval.

18. The abuses which, for the advantage, real or imaginary, of a very few individuals, have thus existed for centuries in the higher branch of this profession appear now to have approached the highest degree of which they are susceptible: preventing the public from having a free choice of their physicians; greatly augmenting the expences incidental to sickness; preventing physicians, with the exception of the favored few, from aspiring, with any chance of success, to offices of dignity or emolument; impeding the progress of science, and infringing upon the rights of the universities.

19. The grounds of all the foregoing assertions your petitioners are prepared to prove, and they trust that, in what they have averred, a sufficient apology is furnished for the present appeal, and a sufficient cause shown why your honorable House should take this most important and long-neglected subject under your early and mature consideration.

20. What precise reforms of the profession may be called for, your petitioners presume not now to mention; they merely submit, most respectfully, to your honorable House, that great amendment is needed in every part of the British dominions. They solicit only inquiry into the defects and abuses of which they complain, and they rely with confidence on your honorable House for granting them, as the result of that inquiry, such relief as your honorable House, in its wisdom, may deem meet.

21. Your petitioners venture only to suggest, that a parliamentary committee of inquiry would, in their opinion, be the most effectual means of eliciting and verifying the necessary information; and they respectfully submit that the importance of the subject, whether as it regards the welfare of the public or the interests of an useful and much aggrieved profession, fully entitles it to the grave examination which they solicit.

And your petitioners, &c.

All physicians, whether in town or country, who may approve the principles here set forth, and desire to contribute their efforts towards obtaining the objects sought, are requested to communicate their sentiments, without delay, to the Faculty of Physic in London, (post free,) to the care of Messrs. T. and G. Underwood, 32, Fleet-street; Messrs. Callow and Wilson, Princes-street, Soho; or Messrs. Burgess and Hill, Great Windmill-street, Piccadilly. And it is further most earnestly requested, that they take an early opportunity of making the members of parliament, magistrates, and other influential persons in their respective neighbourhoods, acquainted with the real bearings of the important questions at issue, which have recently undergone such full and able discussion in the Medical and other Journals of the metropolis, as to have been rendered perspicuous to all who have had an opportunity of attending sufficiently to the subject. For the convenience of those who may wish to suggest improvements, the paragraphs of the proposed petition are numbered.

### METEOROLOGICAL JOURNAL,

*From November 20th, to December 20th, 1827.*

By Messrs. HARRIS and Co. Mathematical Instrument Makers, 50, High Holborn.

| November | Rain gauge. | Moon. | Thermom. | | | Barometer. | | De Luc's Hygrom. | | Winds. | | Atmospheric Variations. | | |
|---|---|---|---|---|---|---|---|---|---|---|---|---|---|---|
| | | | 9 A.M. | MAX. | MIN. | 9 A.M. | 10 P.M. | 9 A.M. | 10 P.M. | 9 A.M. | 10 P.M. | 9 *a.m.* | 2 *p.m.* | 10 *p.m.* |
| 20 | | | 46 | 50 | 41 | 29.96 | 29.97 | 100 | 93 | W | NW | Fair | Fair | Fair |
| 21 | Rain gauge frozen. | | 42 | 43 | 29 | 30.08 | 30.02 | 86 | 84 | NE | NNE | — | — | — |
| 22 | | | 30 | 36 | 25 | 29.77 | 29.65 | 83 | 86 | N | NNW | — | — | Snow |
| 23 | | | 26 | 36 | 27 | 29.55 | 29.51 | 86 | 89 | NW | N | — | — | Fair |
| 24 | | | 30 | 34 | 28 | 29.67 | 29.88 | 88 | 88 | N | N | — | — | Snow |
| 25 | | ☽ | 33 | 39 | 37 | 29.83 | 30.21 | 96 | 98 | WSW | WSW | — | Rain | Cloudy |
| 26 | | | 42 | 45 | 34 | 30 20 | 30.25 | 98 | 100 | WSW | W | Foggy | Cloudy | Sleet |
| 27 | | | 34 | 45 | 34 | 30.22 | 30.12 | 100 | 98 | W | SW | — | — | Fine |
| 28 | | | 36 | 45 | 41 | 29.92 | 29.70 | 98 | 87 | SW | S | Cloudy | — | Cloudy |
| 29 | | | 44 | 48 | 43 | 29.24 | 29.38 | 100 | 98 | NW | NW | Rain | — | Fair |
| 30 | | | 46 | 51 | 47 | 29.36 | 29.35 | 96 | 98 | NW | S | Fair | — | Cloudy |
| Dec. | | | | | | | | | | | | | | |
| 1 | | | 48 | 53 | 44 | 29.01 | 28.96 | 86 | 87 | WSW | WNW | Cloudy | — | Sleet |
| 2 | | | 45 | 47 | 43 | 29.00 | 29.27 | 90 | 96 | NW | ENE | — | Sleet | — |
| 3 | | ○ | 45 | 48 | 44 | 29.55 | 29.71 | 96 | 86 | ENE | W | — | Fair | Cloudy |
| 4 | | | 50 | 56 | 49 | 29.66 | 29.71 | 97 | 93 | W | WNW | — | — | — |
| 5 | | | 51 | 56 | 47 | 29.95 | 29.73 | 95 | 95 | W | W | — | — | — |
| 6 | | | 47 | 49 | 36 | 29 68 | 30.00 | 80 | 85 | WNW | NW | — | — | — |
| 7 | | | 39 | 50 | 45 | 30.14 | 29.80 | 90 | 85 | WNW | NW | — | — | — |
| 8 | | | 46 | 47 | 35 | 29.77 | 29.86 | 88 | 90 | NW | NW | Fair | — | Fair |
| 9 | | | 40 | 55 | 51 | 29.79 | 29.55 | 90 | 98 | W | NW | — | — | Sleet |
| 10 | | | 54 | 55 | 45 | 29.36 | 29.24 | 98 | 98 | SW | SW | Cloudy | Sleet | — |
| 11 | | ☾ | 45 | 53 | 41 | 29.39 | 29.30 | 86 | 93 | WSW | SW | — | Cloudy | Cloudy |
| 12 | | | 44 | 46 | 42 | 29.25 | 29.21 | 97 | 100 | ESE | SE | DenseF. | — | Rain |
| 13 | | | 42 | 45 | 39 | 29.37 | 29.57 | 97 | 90 | WNW | SW | Cloudy | — | Cloudy |
| 14 | | | 44 | 52 | 43 | 29.35 | 29.34 | 92 | 98 | SSW | W | Rain | Rain | — |
| 15 | | | 47 | 55 | 44 | 29.57 | 29.46 | 98 | 98 | WSW | SW | Fair | — | Rain |
| 16 | | | 44 | 51 | 38 | 29.62 | 29.92 | 92 | 90 | W | WNW | — | — | Fine |
| 17 | | | 42 | 49 | 48 | 30.08 | 29.96 | 90 | 90 | W | WSW | — | Fair | Fair |
| 18 | | ● | 51 | 55 | 49 | 29.68 | 29.57 | 97 | 98 | WSW | WSW | Rain | — | Rain |
| 19 | | | 53 | 55 | 46 | 29.49 | 29.46 | 98 | 98 | WSW | WSW | — | Rain | — |

